# Workshops for developing written exam questions go online: appropriate format according to the participants

**DOI:** 10.3205/zma001413

**Published:** 2021-01-28

**Authors:** Wilma Anschuetz, Felicitas Wagner, Patrick Jucker-Kupper, Sören Huwendiek

**Affiliations:** 1 Universität Bern, Institut für medizinische Lehre, Bern, Switzerland

**Keywords:** workshop, online, questions development, multiple choice, medical

## Abstract

**Background: **The Corona pandemic has made it difficult to conduct face-to-face events, which is why two workshops planned for the development of multiple choice (MC) questions were conducted online. Whether the online format is suitable for MC question development has not yet been described to our knowledge.

**Questions: **The study aimed to answer the following questions from the perspective of the participants: How are the two online workshops evaluated in terms of their implementation? Are these online workshops suitable for developing MC questions? Is the online or face-to-face format preferred? As a measure of efficiency, it was examined whether the expected question output (standard of comparable face-to-face workshops) was achieved in the online workshops.

**Methods: **In May and June 2020, two online workshops with a total of 24 participants were conducted for Swiss professional societies with SWITCHinteract. The participants’ feedback was collected via an anonymous online survey with 21 questions.

**Results: **88% of the participants took part in the voluntary online survey. The participants were satisfied with the implementation and found the online format suitable. The majority of the participants did not show a preference for a certain format (online vs. face-to-face), although in case of a format preference the online format was indicated more often. The expected question output was exceeded in both workshops. Technical aspects were most frequently cited as requiring improvement.

**Conclusion: **Based on the results, online workshops for MC question development can be considered as a resource-saving and efficient alternative to face-to-face workshops. Increased use and optimization of online tools could further facilitate implementation and influence the format preference.

## Zusammenfassung

**Hintergrund: **Während der Corona-Pandemie wurde die Durchführung von Präsenzveranstaltungen erschwert, weshalb zwei geplante Präsenz-Workshops zur Erstellung von Multiple Choice (MC)-Fragen online durchgeführt wurden. Ob sich das Online-Format für die MC-Fragenerstellung eignet ist unseres Wissens bisher nicht beschrieben.

**Fragestellungen: **Die Studie hatte das Ziel, folgende Fragen aus Sicht der Teilnehmenden (TN) zu beantworten: Wie werden die zwei Online-Workshops hinsichtlich der Durchführung bewertet? Eignen sich diese Online-Workshops zur Erstellung von MC-Fragen? Wird das Online- oder Präsenz-Format bevorzugt? Als Mass für die Effizienz wurde betrachtet, ob der erwartete Fragenoutput (Standard von vergleichbaren Präsenz-Workshops) in den Online-Workshops erreicht wurde.

**Methoden: **Im Mai und Juni 2020 wurden zwei Online-Workshops mit insgesamt 24 TN für Schweizer Fachgesellschaften mit SWITCHinteract durchgeführt. Die Rückmeldungen der TN wurden per anonymer Online-Umfrage mit 21 Fragen erhoben.

**Ergebnisse: **88% der TN nahmen an der freiwilligen Online-Umfrage teil. Die TN waren zufrieden mit der Durchführung und empfanden das Online-Format als geeignet. Die Mehrheit der TN zeigte keine Präferenz für ein bestimmtes Format (Online vs. Präsenz), wobei jedoch im Fall einer Format-Präferenz häufiger das Online-Format angegeben wurde. Der erwartete Fragenoutput wurde in beiden Workshops übertroffen. Als verbesserungswürdig wurden am häufigsten technische Aspekte angegeben.

**Schlussfolgerung: **Anhand der Ergebnisse können Online-Workshops zur MC-Fragenerstellung als ressourcenschonende und effiziente Alternative für Präsenz-Workshops betrachtet werden. Die zunehmende Anwendung und Optimierung von Online-Tools könnte die Umsetzung weiter erleichtern und die Format-Präferenz beeinflussen.

## 1. Introduction

The development of multiple choice (MC) exam questions is a demanding process that can be efficiently carried out in structured face-to-face workshops [1]. During the corona pandemic, face-to-face workshops were made more difficult due to necessary safety concepts, which is why planned in-person workshops were conducted online. Publications on other online events show that they are well suited for knowledge transfer [2], [3] and offer advantages such as flexibility, as well as time and cost savings [2], [4], [5], which results in a high willingness to participate and acceptance [6], [7], [8], [9]. To our knowledge, there are no studies that have investigated the suitability of an online workshop for MC question development.

## 2. Research questions

This study aimed to answer the following questions from the perspective of the participants:

1. How are the two online workshops evaluated in terms of implementation?
2. Are these online workshops suitable for developing MC questions?
3. Is an online or face-to-face format preferred?

In terms of efficiency, the following was examined:

1. Is the expected question output in the online workshops achieved?

## 3. Methods

### 3.1. Procedure

In May and June 2020, two independent question workshops for Swiss professional societies with a total of 24 participants were conducted online. The same question output was expected as for a face-to-face workshop (see table 1 (Tab. 1)). 

The workshops were conducted with SWITCHinteract [https://www.switch.ch/interact/], an Adobe Connect based online seminar tool, which is recommended for webinars with features such as video conferencing, breakout rooms and screen-share [4]. In the run-up to the seminar, participants were given instructions on how to develop MC questions and set up SWITCHinteract (including test login). The workshops were conducted based on that of face-to-face workshops (see table 2 (Tab. 2)).

#### 3.2. Survey

An anonymous online questionnaire (Questback Unipark, [https://www.unipark.com/]) was used to ask the participants about the implementation, suitability and preference of the workshop format. The questionnaire contained five demographic, 17 closed questions with a 6-point Likert scale or matching nominal or ordinal scale and four optional free text questions. The questionnaire was newly developed for this study. The participants received a link to the voluntary survey after the workshop.

#### 3.3. Analysis

All results are presented descriptively. Due to the small number of cases no statistical comparisons were calculated. The free text comments were analyzed with regard to recurring topics. In the following, the results are only presented for those 16 questions that are relevant for the answers to the research questions.

## 4. Results

The response rate was 88% (21/24 participants).

### 4.1. Demography

38% of the participants had previous experience as question authors, 14% with question workshops (see table 3 (Tab. 3)).

#### 4.2. Implementation 

Overall, the participants rated the implementation (organization, schedule, support, content) as good (see table 4 (Tab. 4)).

#### 4.3. Suitability

On average, the participants were (rather) skeptical in advance whether the virtual implementation was suitable. In the end, the online format and SWITCHinteract were considered suitable on average. The majority of the participants were happy about the elimination of the travel time, which would have added up to 46 hours (see table 5 (Tab. 5)). External factors disturbed 43% of participants moderately (see table 6 (Tab. 6)). In the free text questions a few aspects were mentioned several times (see table 7 (Tab. 7)).

#### 4.4. Preference 

**Figure 1:**
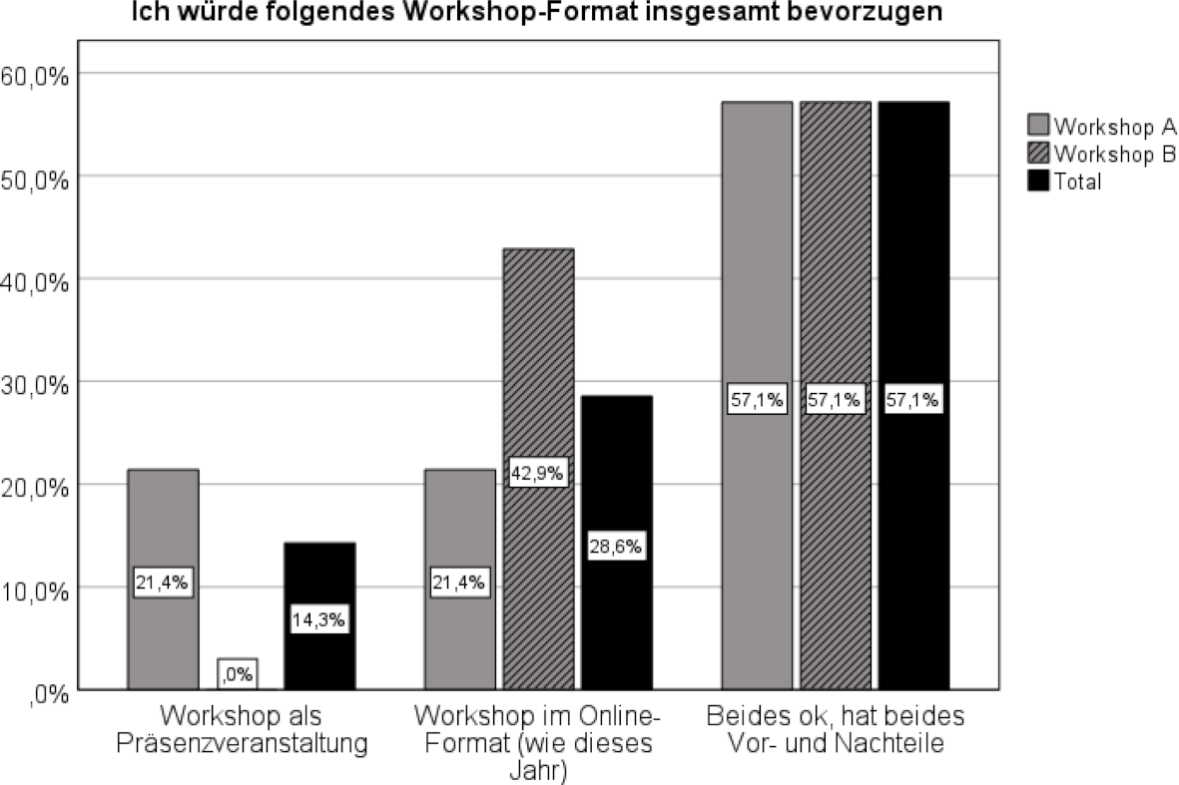
Preferred workshop format. All data in percent (rounded).

There was no clear preference for a workshop format. The majority of participants saw advantages and disadvantages in both formats (see figure 1 (Fig. 1)).

#### 4.5. Question output

In both workshops the expected question output was exceeded (see table 8 (Tab. 8)).

## 5. Discussion

This study examined the suitability of online workshops for the development of MC questions. 

The participants were satisfied with the implementation and found the online format and the tool used to be suitable, although there were some online skeptics in the run-up to the workshop. A total of 46 hours of travel time was saved, which the majority of participants found to be an advantage. The majority (57%) of the participants showed no preference, 29% preferred an online format and 14% a face-to-face format. The expected question output was exceeded in both workshops. External factors disturbed 43% of the participants moderately. Technical problems were most frequently mentioned as an aspect in need of improvement in the free text questions.

With the implementation judged to be good and the online format perceived as suitable, questions 1 and 2 can be answered positively. Together with the high question output, this indicates that the procedure, content and expected output of the face-to-face workshop were transferable to the online format. The reported time savings and related cost and CO_2_ savings were also described in other publications [2], [6], [7]. 

Our results lead to the conclusion that technical aspects should be optimally prepared (detailed advance information on the online tool, test login, function of microphone/camera/screen share, stable Internet, quiet environment) in order to minimize disruptive factors as much as possible.

The majority of the participants did not have a clear format preference, but more participants who stated a preference preferred the online format, which confirms the results of [2]. 

Limitations of this study are the small number of participants and the fact that it cannot be assessed at this point whether the quality of the questions (performance in exams) is comparably high. 

Based on the available results, online workshops for the development of MC questions can be regarded as a resource-saving and efficient alternative to face-to-face workshops. In addition to optimal technical preparation, increased use and optimization of online tools could facilitate implementation in the future and influence format preference.

## Acknowledgement

We would like to thank all participants for their commitment before and during the workshops and for participating in the survey.

## Competing interests

The authors declare that they have no competing interests. 

## Figures and Tables

**Table 1:**
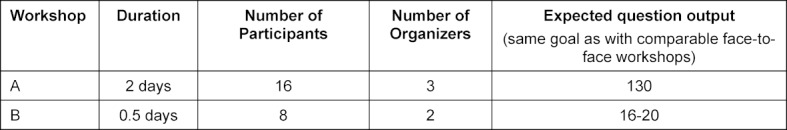
Implemented workshops

**Table 2:**
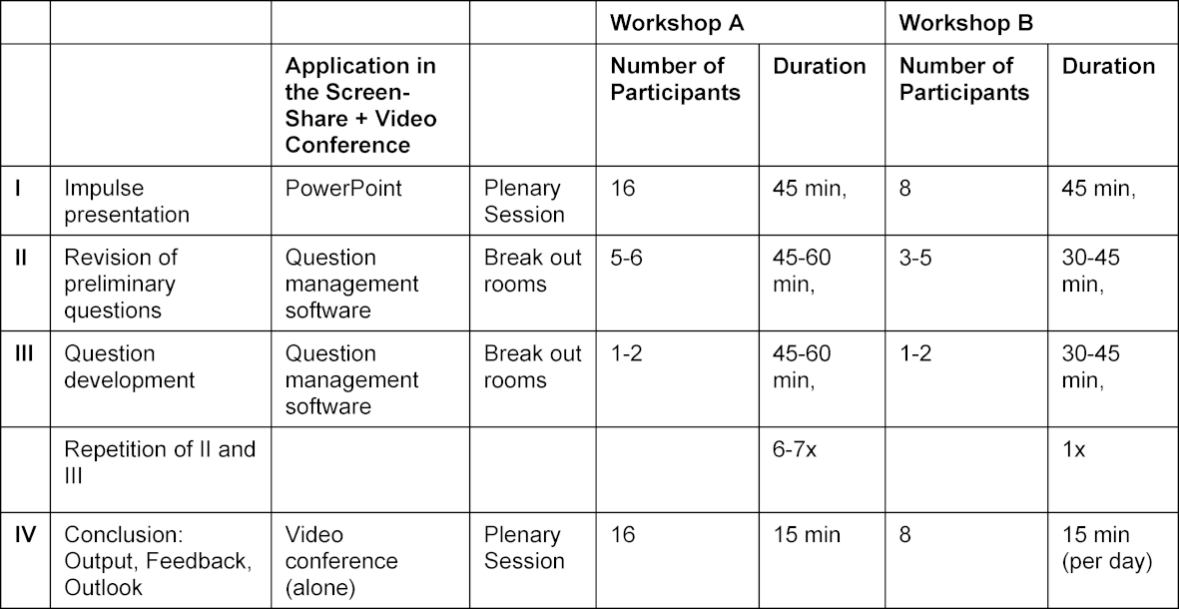
Schedule of the workshops

**Table 3:**
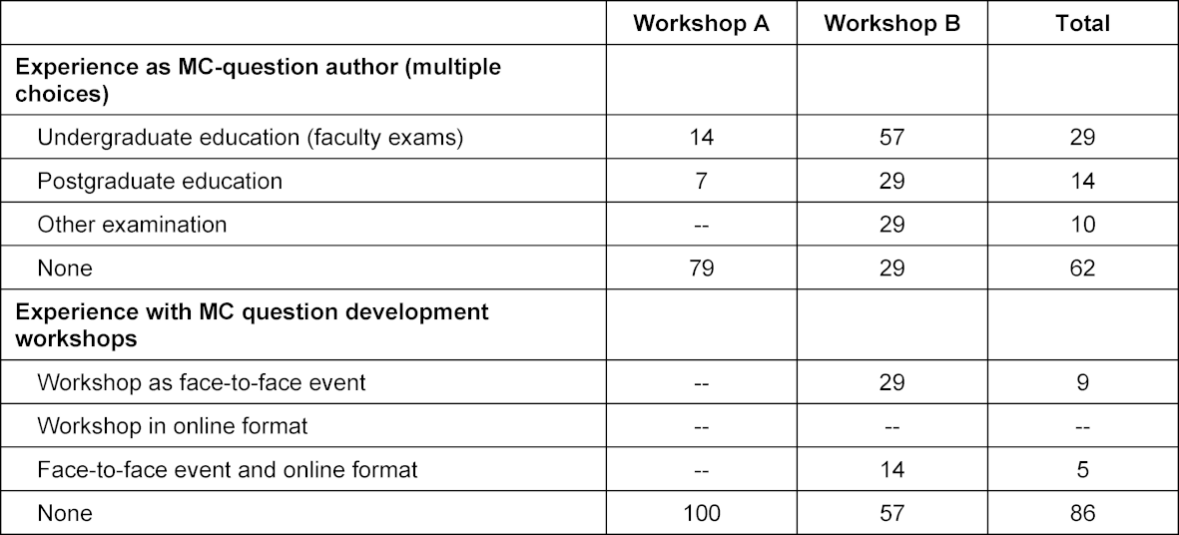
Demographic data of the participants. All data in percent (rounded).

**Table 4:**
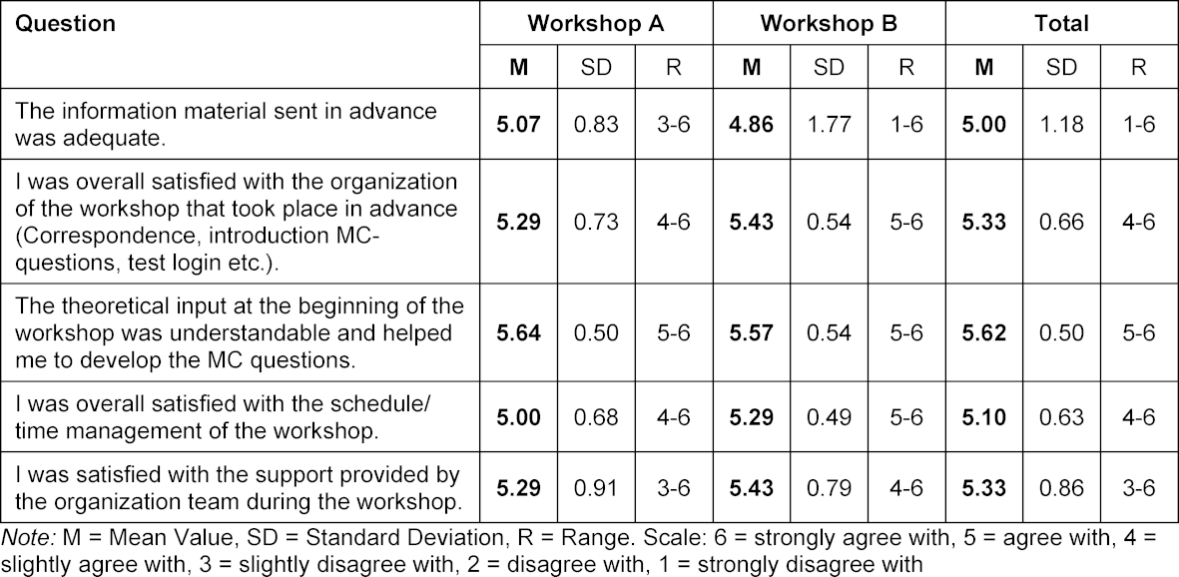
Assessment of implementation (organization, schedule, support and content)

**Table 5:**
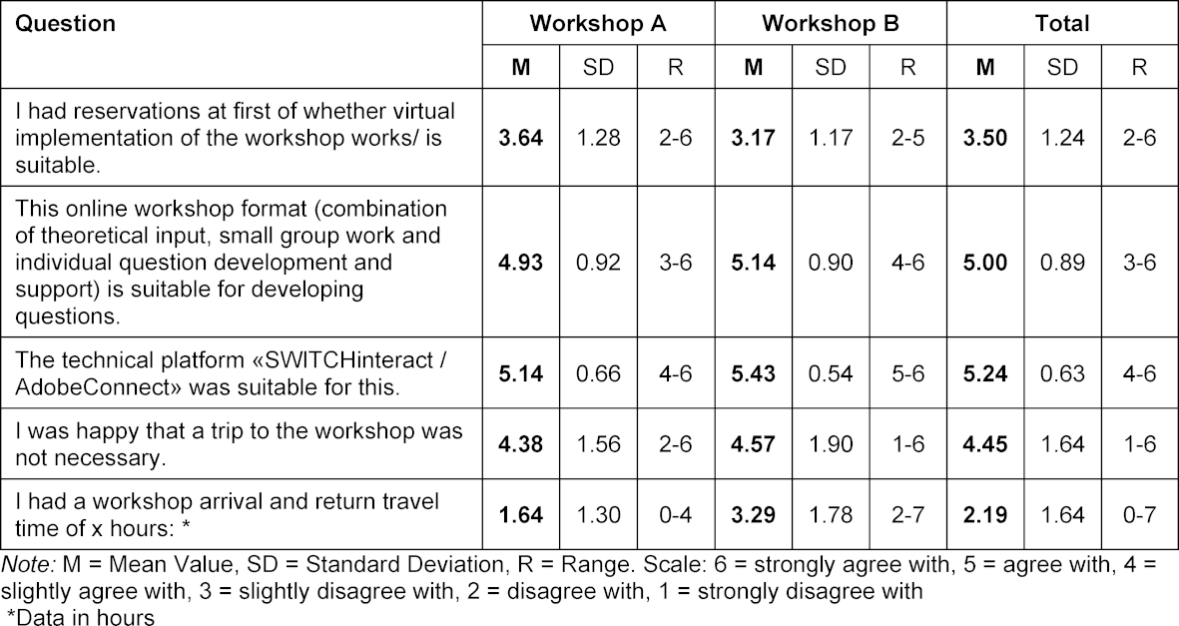
Suitability of the workshop format

**Table 6:**
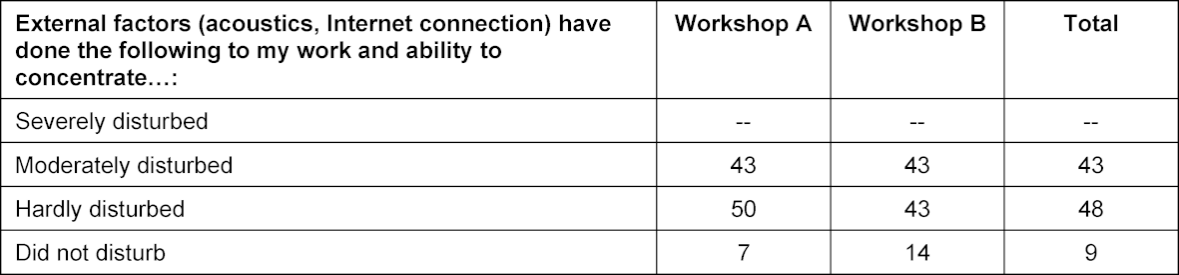
Disturbance by external factors. All data in percent of the participants (rounded)

**Table 7:**
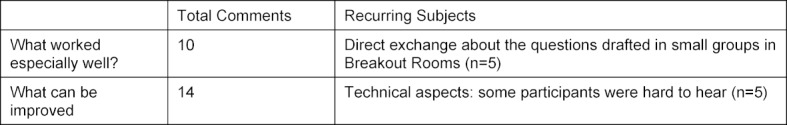
Recurring subjects with regard to the research questions. Only aspects are listed, which were mentioned by at least two people.

**Table 8:**
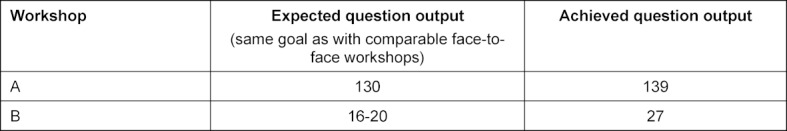
Question output
